# Rheological and Viscoelastic Properties of Chitosan Solutions Prepared with Different Chitosan or Acetic Acid Concentrations

**DOI:** 10.3390/foods11172692

**Published:** 2022-09-03

**Authors:** Paulo José do Amaral Sobral, Gebremedhin Gebremariam, Federico Drudi, Ana Cristina De Aguiar Saldanha Pinheiro, Santina Romani, Pietro Rocculi, Marco Dalla Rosa

**Affiliations:** 1 Department of Food Engineering, Faculty of Animal Science and Food Engineering, University of São Paulo, Av. Duque de Caxias Norte, 225, Pirassununga 13635-900, SP, Brazil; 2 Food Research Center (FoRC), University of São Paulo, Rua do Lago, 250, Semi-Industrial Building, Block C, Sao Paulo 05508-080, SP, Brazil; 3 Department of Agricultural and Food Sciences, *Alma Mater Studiorum* University of Bologna, Campus of Food Science, 47521 Cesena, Italy; 4 Interdepartmental Centre for Agri-Food Industrial Research, *Alma Mater Studiorum* University of Bologna, Campus of Food Science, 47521 Cesena, Italy

**Keywords:** polysaccharide, solubility, consistency index, index flow behavior, storage modulus, loss modulus

## Abstract

Chitosan (Ch) is a partially crystalline biopolymer, insoluble in pure water but soluble in acid solutions. It has attracted interest from researchers to prepare solutions using different acid types and concentrations. This research aims to study both the effect of chitosan (Ch) or acetic acid (Ac) concentrations, at different temperatures, on rheological and viscoelastic properties of Ch solutions. To study the effect of Ch, solutions were prepared with 0.5–2.5 g Ch/100 g of solution and Ac = 1%, whereas to study the effect of Ac, the solutions were prepared with 2.0 g of Ch/100 g of solution and Ac = 0.2–1.0%. Overall, all analyzed solutions behaved as pseudoplastic fluid. The Ch strongly affected rheological properties, the consistency index (*K*) increased and the index flow behavior (*n*) decreased as a function of Ch. The activation energy, defined as the energy required for the molecule of a fluid to move freely, was low for Ch = 0.5%. The effect of Ac was less evident. Both *K* and *n* varied according to a positive and negative, respectively, parabolic model as a function of Ac. Moreover, all solutions, irrespective of Ch and Ac, behaved as diluted solutions, with G” > G’. The relaxation exponent (n”) was always higher than 0.5, confirming that these systems behaved as a viscoelastic liquid. This n” increased with Ch, but it was insensitive to Ac, being slightly higher at 45 °C.

## 1. Introduction

Chitosan (Ch) is a partially deacetylated biopolymer of acetyl glucosamine, obtained after the alkaline treatment for N-deacetylation of the chitin [1]. Chitosan has two functional groups (OH and NH_2_), and because of the latter, it is a positive polyelectrolyte when solubilized in water at a pH below 6.5 [2,3]. As powder, it is a partially crystalline biopolymer that is insoluble in pure water [2]. Chitosan has attracted the interest of researchers in the field of agriculture, who are searching for a matrix to encapsulate fertilizer and then improve its effectiveness [4]; in electrochemistry, where researchers are searching to produce biodegradable material for energy storage, due to its polyelectrolyte character when solubilized [3,5]; in the field of biomaterials [6,7], including biomembranes [8], because it is non-toxic and biocompatible with the human physiological system and can be easily chemically modified; and in the domain of edible/active film technology because it has a good film-forming property and has an intrinsic antimicrobial property [1,9] with improved antimicrobial activity by the incorporation of nanoparticles [10].

Usually, these materials are prepared by wet techniques which are usual for the preparation of Ch solutions. To prepare these solutions, the most common method is to disperse 1% of Ch into 1% of acid acetic (Ac) solution, but some authors used different conditions (Table 1) and, usually, they maintain these solutions under mixing for 24 h to reach complete solubilization. By adding a plasticizer in these solutions, film-forming solutions (FFS) were prepared. These FFS, poured in a convenient support, produced films after drying [11].

It is important to remark that the conditions shown in Table 1 do not constitute independent variables in the respective experiments, therefore a lack of information on the effect of the Ch and/or Ac on properties of the final product must be expected. To begin with, it can be interesting to study the effect of these conditions on rheological properties of Ch solutions. It is well known that rheological tests (e.g., by steady state or dynamic tests) of biopolymers-based solutions can give insights on the structure–property relations between biopolymer and solvent [28,29,30]. Moreover, specifically to film technology, steady state test results can help to optimize the film production, especially in the casting and spreading techniques since rheology studies interplay between flow and material properties [29]. The viscosity of FFS must be suitable to allow a good casting on the support, which means that FFS must flow under the action of gravity but avoid sagging when FFS is applied by a spreader [18]. However, a high-viscosity FFS would make it difficult to eliminate air bubbles, provoking the production of a porous matrix when dried to produce film [18,29].

The viscoelastic properties are also important because they can provide information directly related to the conformation of the macromolecules and to the molecular relaxation phenomenon [31]. In practical terms, there are two types of tests to determine the viscoelastic properties: static and dynamic tests [32,33]. In the static tests, a constant deformation (or stress) is imposed, and the variation of the stress (or the deformation) must be observed during the progress of time. The first test is called stress relaxation and the second one, creep compliance [32,33]. In these cases, the model utilized in data treatment establishes the viscoelastic properties [31,32,33]. During the dynamic (or oscillatory) test, a sinusoidal variation of the deformation (or stress) is imposed on the material and a variation of the stress (or deformation) necessary must be observed, which must also oscillate according to a sine curve but with a phase displacement of 0 to π/2 in relation to the deformation (or stress). If the material is purely elastic (Hookean) or liquid (Newtonian), the phase displacement will be 0 or π/2, respectively [32,33].

There are several works on rheological properties of FFS based on Ch, pure or blended with other biopolymers [12,14,18,19], but few papers on Ch solutions. El-Hefian et al. [21] studied the effect of the temperature (20–50 °C) and Ch (2–10 g/L) on the viscosity of Ch solutions (Table 1) and observed that these solutions behaved like pseudoplastic fluids and that viscosity decreased as a function of the temperature and increased as a function of Ch. Similarly, Hwang and Shin [27] and Alvarado et al. [23] also observed a positive effect of Ch on the viscosity of Ch solutions. Martínez-Ruvalcaba et al. [26] determined G’ and G” as a function of frequency (0.3–60 rad/s), at 25, 30, 37, and 45 °C and 0.5, 1.0, 1.5, and 2.0% of Ch. They observed that both G’ and G” increased as a function of frequency, always with G” > G’, but with a tendency of crossover at a higher frequency. Moreover, they observed that G” increased as a consequence of Ch increasing. In addition, Tovar et al. [22] used both tests (steady state and dynamic) to study the effect of (lactic and acetic) acid solutions on the rheological properties at several Ch. They observed that rheological properties determined in steady state tests varied as a function of Ch in different manners depending on the acid type and that, at fixed Ch, lactic acid solutions had higher G’ and G” than acetic acid solutions. From these works, it is clear that the effect of different acid concentrations (or different solution pH) has not yet been studied.

In this sense, the objectives of this work were to study the effect of (i) Ch concentration at three temperatures (25, 35, and 45 °C) and (ii) acetic acid concentration of 2% of Ch (25 and 45 °C) on rheological properties determined by steady state (flow curves), and by dynamic tests, specifically by strain and frequency sweep tests. The temperatures were chosen considering that 25 °C corresponds to the room temperature and 45 °C is close to the Ch solutions processing temperature.

## 2. Materials and Methods

### 2.1. Material

Three batches of Ch were used without further purification in this study: a low viscosity and diacetyled at ≥75%, 50494-100G, Lot #BCBT1850 from Sigma-Aldrich, Co. (Darmstadt, Germany) was used in the study of the effect of Ch; a low molecular weight, 448869-50G, Lot #BCCD0403 from Sigma-Aldrich, Co. (Hafnarfjordur, Iceland) was used to study the effect of acid acetic (Ac) concentration; and another low molecular weight, 448869-250G, Lot #SLBJ5775V from Sigma-Aldrich, Co. (Darmstadt, Germany), was used to prepare solutions for freeze-drying. The Ac (≥99.8%) was from Sigma Aldrich, Co. (Darmstadt, Germany).

### 2.2. Preparation of Chitosan Solutions

Ch solutions were prepared for two experiments: (i) to study the effect of the Ch, different Ch solutions were prepared with 0.5, 1.0, 1.5, 2.0, and 2.5 g of Ch/100 g of solution, dispersed in 1% Ac solution; (ii) to study the effect of the Ac, different solutions were prepared with 2.0 g of Ch/100 g of solution, dispersed in 0.0, 0.2, 0.4, 0.6, 0.8, and 1.0% (*w*/*w*) Ac solutions, being that a solution prepared with 0% of Ac was produced only for characterization of freeze-dried samples. This concentration of 2% of Ch was chosen for (ii) because it corresponds to a film-forming solution that is not so viscous to render casting very difficult or so diluted to demand a high amount of FFS on Petri plates.

Ch solutions were prepared according to Benoso et al. [12], with some modifications. Briefly, these solutions were prepared by dispersing Ch, duly weighted in an analytical balance (ABJ Kern, Balingen, Germany), into a convenient initial amount of water, at 40 °C, under mixing, followed by using a convenient amount of the Ac to attain the desired final concentration that was added, always under mixing. Finally, the total amount of the solution was completed by adding water, also under mixing. After preparation, all solutions were submitted to an ultrasound treatment for 5 min, using an ultrasound bath (Elmasonic, Singen, Germany) to eliminate bubbles. Rheology tests were carried out immediately after solutions preparation. The pH of these solutions was also measured using a digital pH meter (Easyfive Plus, Mettler Toledo, Columbus, OH, USA).

Moreover, aiming to study the effect of the Ch or Ac in Ch solubilization, similar solutions were prepared, added in Kitasato flasks, frozen by quench cooling in liquid nitrogen, freeze-dried in a freeze-dryer (FD 1.0-60E, Heto-Holten A/S, Allerod, Denmark) coupled to a vacuum pump (RZ 6, Vacuubrand, Wertheim, Germany), and submitted to analyses by scanning electron microscopy, Fourier transform infrared spectroscopy, and X-ray diffraction.

### 2.3. Freeze-Dried Chitosan Characterizations

#### 2.3.1. Scanning Electron Microscopy

Samples were analyzed by scanning electron microscopy (SEM) using a Hitachi Tabletop Microscope TM3000 (Hitachi Ltd., Tokyo, Japan). They were mounted on copper stubs and analyzed with an accelerating voltage of 5 kV, without any previous preparation [1].

#### 2.3.2. Fourier Transform Infrared Spectroscopy (FTIR)

Samples were analyzed using an FTIR spectrophotometer (PerkinElmer Spectrum One, Stuttgart, Germany) from the wavenumber 4000 to 500 cm^−1^ with a resolution of 4 cm^−1^, and 32 scans, using the universal attenuator total reflectance (UATR) accessory [34]. The FTIR spectra were taken in transmittance mode.

#### 2.3.3. X-ray Diffraction

X-ray diffractograms (XRD) of samples were obtained using an X-ray diffractometer (MiniFlex 600, Rigaku, Tokyo, Japan), operating at 40 kV and 15 mA (Cu Kα 1, λ = 1.54056 Å radiation). Spectra were recorded at 25 °C with angles from 2θ = 2 to 70° at a rate of 2°/min [35].

### 2.4. Chitosan Solutions Characterizations: Rheological Tests

Ch solutions were analyzed by rheological tests carried out in a modular compact rheometer (MCR102, Anton Paar, Graz, Austria) using a plate–plate geometry (50 mm diameter) with a gap of 1 mm. The temperature was controlled by a Peltier system, and water evaporation was avoided by using a solvent trap accessory.

#### 2.4.1. Steady Shear Tests

In the steady shear tests, measurements were performed in the shear rate range of 0–100 s^−1^ [12], at 25, 35, and 45 °C. No induced instabilities nor material ejection were observed; and thixotropic behavior was not observed in previous essays.

#### 2.4.2. Dynamic Tests: Frequency Sweep Tests

Initially, strain sweep tests were performed to define the extent of the linear viscoelastic region (LVR), with strain ranging from 0.01–100%, 1 Hz of frequency, at 25 and 45 °C. The behavior of the storage modulus (G’) and the loss modulus (G”) as a function of strain, which was influenced by Ch and Ac, as well as the temperature, allowed the definition at 5% to be the better condition for all Ch solutions measurements, guaranteeing it to be inside of the LVR. Then, the Ch solutions were subjected to sweep tests from 0.1 to 50 Hz, at 25 and 45 °C. The results of these tests were presented as G’ and G” as a function of frequency [28].

### 2.5. Data Analysis

All data were generated by the software of the rheometer and analyzed using the software Excel^®^2020, including for all fittings. The crystallinity of freeze-dried samples was calculated using the software Origin2020.

## 3. Results and Discussions

Preparing a chitosan (Ch) solution is not simple. Adding the Ch powder directly into an acetic acid (Ac) solution may produce agglomerates which will stay insolubilized, demanding a lengthy time preparation with mixing. Otherwise, the final Ch concentration in the resulting solution will be different from that previously planned.

All solutions were transparent (Figure S1) and had a very visible effect of Ch: more concentrated solutions were thicker and presented more air bubbles than those less concentrated. In addition, the effect of Ac concentration was visible, with samples becoming less thick, presenting fewer bubbles, and displaying less transparency as a consequence of the reduction of Ac (Figure S2). Because of that, these solutions were freeze-dried for characterizations.

It is interesting to note that the pH of Ch solutions varied with both studied concentrations. While the pH increased linearly (Equation (1), Table 2) from 3.70 to 4.61 when Ch increased from 0.5 to 2.5%, it decreased also linearly from 5.90 to 4.47 as a function of the increasing Ac from 0.2 to 1.0% (R^2^ > 0.97, in both cases) (Table 2). Similar results were obtained by Tovar et al. [22], who prepared solutions (Ac not described) with Ch = 1, 2, and 3% and determined pH= 3.50, 3.79, and 3.98, respectively.

*Y* = *A* + *BX* (1)

When Ch was dissolved in aqueous Ac solutions, a cationic polymer was formed due to protonation of amino groups on the C-2 position of the pyranose ring, promoting its own solubilization [6,36]. According to Rinaudo et al. [36], the complete Ch solubilization is obtained when the degree of protonation is around 0.5.

Considering that the Ac is a weak acid, the equilibrium, represented by Equations (2) and (3), must be considered [36].

CH_3_COOH + H_2_O ↔ CH_3_COO^−^ + H_3_O^+^ (2)

Ch-NH_2_ + H_3_O^+^ ↔ Ch-NH_3_^+^ + H_2_O (3)

Therefore, for a constant Ac, when the Ch increased, more H_3_O^+^ was needed to protonate Ch-NH_2_ (Equation (3)), displacing the acid equilibrium (Equation (2)) towards the right, thus increasing pH. In contrast, for a constant Ch] when the Ac increased, more Ch-NH_2_ was protonated, but the Ac remained high, leading to a lower pH.

Benoso et al. [12] prepared Ch solutions with different pH using Ac solutions and verified that their ξ-potential, which measures the net charge of the macromolecule in the solution, was always positive, decreasing linearly from 70 to 38 mV when the pH increased from 3.5 to 6.0 and confirming that the protonation was dependent on pH. Abu-Jdayil et al. [14] observed that the ξ-potential for the Ch solution slightly decreased to almost zero mV by increasing the Ch, as a consequence of the Ch effect on the final pH of solutions prepared with constant Ac.

### 3.1. Freeze-Dried Chitosan Solutions Characterizations

#### 3.1.1. Scanning Electron Microscopy (SEM)

**Figure 1:**
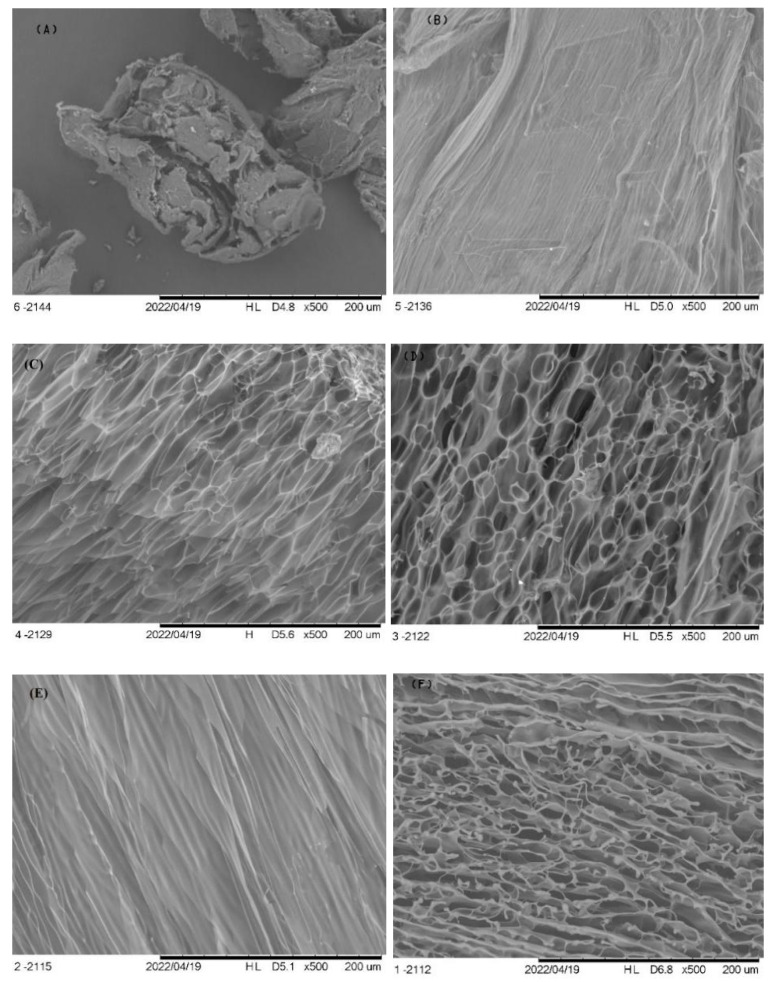
Scanning electron micrography of freeze-dried chitosan solutions prepared with 0.0 (**A**), 0.2 (**B**), 0.4 (**C**), 0.6 (**D**), 0.8 (**E**), and 1.0% (**F**) of acetic acid solutions.

It is well known that Ch is soluble only in acidic media [2]. Thus, evidently, samples prepared with pure water were not solubilized, and the freeze-dried material appeared as particles, like grains, formed by rugous, unevenly broken and fibrillar structures (Figure 1A). No continuous matrix was observed (Figure 1A). Continuous porous matrix, typical of freeze-dried material, was observed for all freeze-dried solutions produced using Ac (Figure 1B–F). Nevertheless, some insolubilized structures, with the same texture of Ch grains, appeared in samples prepared with 0.2% of Ac (Figure 1B). No SEM micrographs on freeze-dried chitosan solution were found in the literature.

#### 3.1.2. Fourier Transform Infrared Spectroscopy (FTIR)

**Figure 2:**
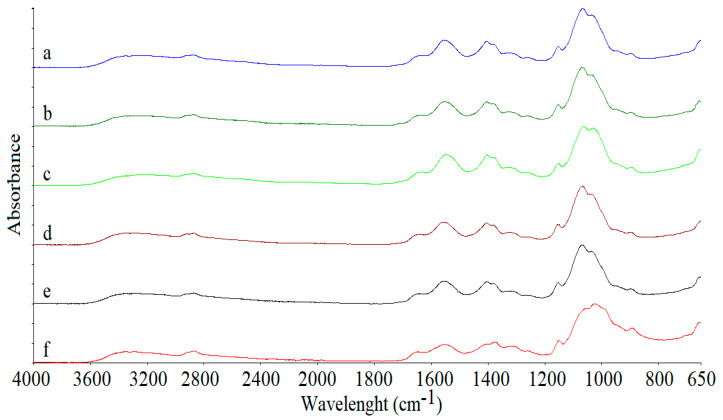
Fourier-transformed infrared spectra of freeze-dried chitosan solutions prepared with 1.0 (**a**), 0.8 (**b**), 0.6 (**c**), 0.4 (**d**), 0.2 (**e**), and 0.0% (**f**) of acetic acid solutions.

FTIR spectra of the freeze-dried Ch solution prepared with pure water must correspond to that of the pure and native Ch (Figure 2f). This spectrum showed a band at 3353 cm^−1^ associated with stretching vibrations of O–H and N–H groups and O–H/N–H interacting with the oxygen of the C=O group, although Pereira et al. [4] have determined this band at 3443 cm^−1^ for native Ch. Following, a band appeared at 2867 cm^−1^ associated with the asymmetrical stretching of -CH [2]. Some signals associated with N-acetyl groups were identified: 1645 cm^−1^, corresponding to the C=O stretching of amide I, visible at 1634 cm^−1^ by Pereira et al. [4], and 1553 cm^−1^, associated with N-H of amide II [20]. The band at 1375 cm^−1^ was associated with the vibrations of the -OH group of the primary alcoholic group [1]. The bands observed at 1405 and 1316 cm^−1^ corresponded to -CH_2_ bonding of methylene groups and methyl groups, respectively. The bands at 1150, 1054, 1023, and 891 cm^−1^ can be attributed to glycosidic linkages and the stretching of C-O-C of the polysaccharide chain [2,20]. The band at 891 cm^−1^ corresponded to the C-H curve out of the ring plan of monosaccharides [1].

Overall, all spectra from samples prepared with Ac (Figure 2a–e) were similar, but they presented some differences in relation to those prepared with water (Figure 2f). In these spectra, the band at 1375 cm^−1^ appeared more important than the precedent, at 1405 cm^−1^, while, for samples prepared with Ac, the band at 1405 cm^−1^ appeared like a peak and that at 1375 cm^−1^ appeared as a shoulder, similar to that observed by Silva-Weiss et al. [19] after analyzing films based on Ch. Considering that these bands were associated with the vibrations of the -OH group of the primary alcoholic group, this change could be due to bond interactions between OH groups from Ch and Ac. The other difference was observed in bands appearing in the spectrum of samples prepared with water at 1054 and 1023 cm^−1^, where this second was the more important signal. These bands, which corresponded to the stretching of C-O-C of the Ch chain [19], shifted to 1066 and 1032 cm^−1^ in spectra of samples prepared with Ac, meaning that the solubilization of Ch was implied in the change of this stretching. Thus, the FTIR analysis was not able to assess changes in the effect of the Ac, or the final pH of the solutions, on Ch solubilization.

#### 3.1.3. X-ray Diffraction

**Figure 3:**
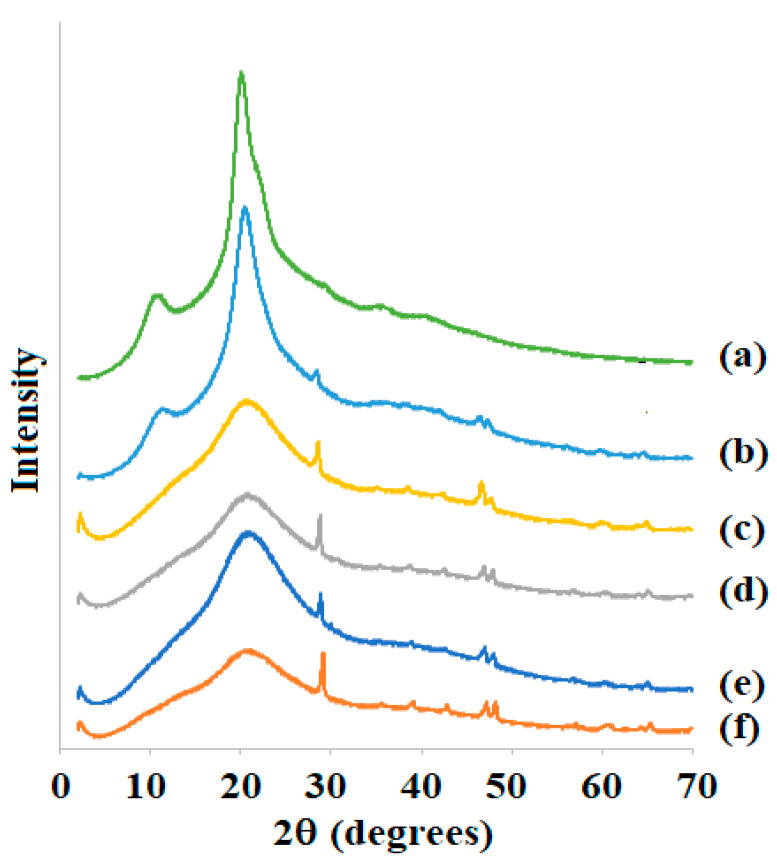
X-ray diffractograms of freeze-dried chitosan solutions prepared with 0 (**a**), 0.2 (**b**), 0.4 (**c**), 0.6 (**d**), 0.8 (**e**), and 1.0% (**f**) of acetic acid solutions.

The X-ray diffraction (XRD) spectrum of the freeze-dried Ch solution prepared with water (Figure 3) presented peaks at approximately 2θ= 11.2° and 20.2°, referring to 020 (crystal 1) and 110 (crystal 2) planes, respectively, typical of Ch [4,37]. The XRD spectrum of samples prepared with 0.2% of Ac (Figure 3b) presented these same peaks, confirming that not all amounts of Ch were solubilized. Moreover, some little peaks appeared at 2θ ≈ 29, 47, and 48°, probably due to the presence of some salts delivered from solubilized Ch fraction, which crystallized during freeze-drying. These peaks appeared for all samples prepared with Ac and were more pronounced for more solubilized samples, i.e., for more important Ac. The XRD spectra of Ch solutions prepared with 0.4–1% of Ac (Figure 3c–f) did not present the first peak, meaning that Ch solubilization dissolved all crystals. In addition, the second peak became the traditional peak of the amorphous portion.

The crystallinity (Ic) of these samples was calculated as the ratio between the peak’s surface and total surface of the XRD spectra. Then, the Ic of the freeze-dried Ch solution prepared with water and 0.2% of Ac were similar (~17%) and higher than those of samples prepared with 4–1% of Ac (Ic ≈ 2.5%). The Ic of these samples was due to the salt crystallinity, as explained above. The Ic of the freeze-dried Ch solution prepared with water was quite lower than that determined by Poerio et al. [37], which analyzed Ch samples produced in laboratory scale and calculated Ic = 53.7%. Indeed, the XRD spectrum determined by Poerio et al. [37] showed a very broad and tall first peak, different from that observed in the present work, thus explaining this high crystallinity.

### 3.2. Rheological Properties of Chitosan Solutions

#### 3.2.1. Steady State Tests: Flow Curves

##### Effect of Chitosan Concentration on Rheological Properties

**Figure 4:**
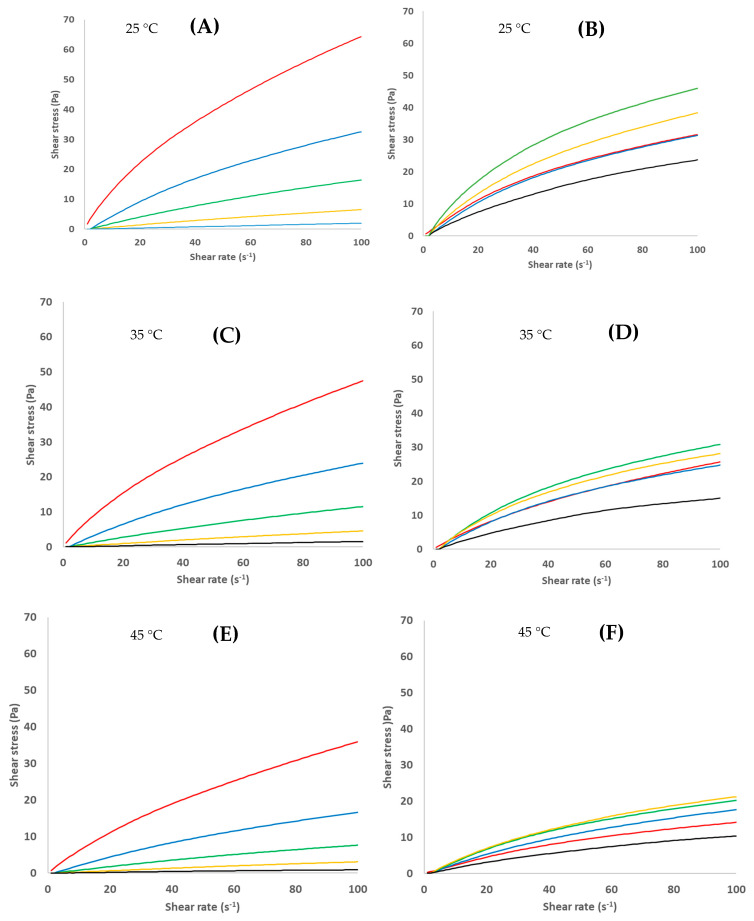
Flow curves of chitosan solutions prepared with several chitosan concentrations (**A**,**C**,**E**): 2.5 (red), 2.0 (blue), 1.5 (green), 1.0 (yellow), and 0.5% (black); and for several acetic acid concentrations (**B**,**D**,**F**): 1.0 (red), 0.8 (blue), 0.6 (green), 0.4 (yellow), and 0.2% (black).

Overall, Ch solutions presented a shear-thinning (pseudoplastic) non-Newtonian behavior for all studied Ch and temperature (T) (Figure 4A,C,E), which means that their viscosity decreased as the shear rate increased, mainly due to the gradual microstructure breakage, usual in colloidal systems, and that interactions between biopolymer–biopolymer were higher than the interactions biopolymer–water [28]. Indeed, the solution less diluted (0.5% of Ch) behaves as Newtonian fluids, meaning that interactions with biopolymer–water prevailed [28]. Similarly, Abu-Jdayil et al. [14], working on the effect of Ch (0.1–3%), observed a non-Newtonian behavior overall, except for Ch ≤ 0.5%.

The effect of Ch on flow curves can be easily observed in Figure 4A,C,E: as the Ch increases, curves get higher. In fact, by increasing Ch, the freedom of movement of the individual chains becomes restricted due to the correspondingly increased number of entanglements [27]. In addition, the effect of temperature (T) can be subjectively observed in Figure 4A,C,E: obtaining lower shear stress values for the same shear applied with the increasing of T due to the increase in thermal motion of the biopolymer with T [21].

Similar pseudoplastic behavior was reported by El-Hefian et al. [21], analyzing Ch solutions with different Ch (2–10 g/L) and T (20–50 °C); by Hwang and Shin [27], for Ch solutions with different Ch (1–2.5%) at 25 °C; by Torres et al. [24], testing Ch solutions with 0.025, 0.03, and 0.035 g/mL at 30 °C; and by Abu-Jdayil et al. [14], for Ch solutions with 0.1–3% of Ch, among others. This same behavior was also observed in works on blends of Ch and starch [16] or gelatin [12] solutions and in FFS based on Ch/gelatin blends [15].

To calculate rheological properties, the power law model (Equation (4)) was fitted to shear stress vs. shear rate data, by non-linear regression (results of fitting are in the Table S1).

(4) $$\tau =K\;{\dot{\gamma }}^{n}$$

where *τ* is the shear stress (Pa), $\dot{\gamma }$ is the shear rate (s^−1^), and *K*, the consistency index (Pa.s*^n^*), and *n*, the index flow behavior (dimensionless), are the rheological properties from the power law model.

**Figure 5:**
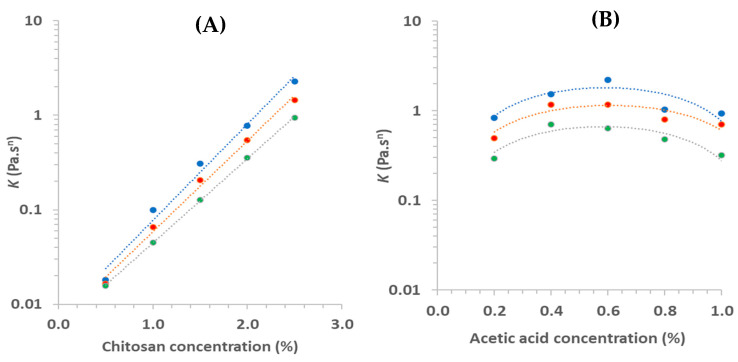

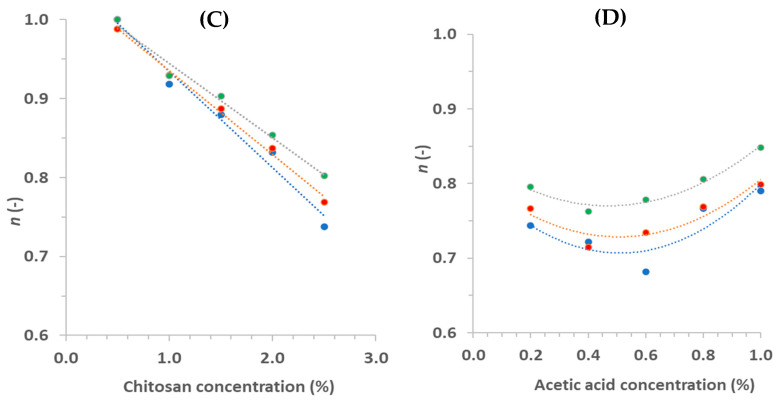
Consistency index (*K*) and index flow behavior (*n*) as a function of the chitosan concentration (**A**,**C**) and of the acetic acid (**B**,**D**), at 25 (blue), 35 (red), and 45 °C (green).

Overall, the *K* varied between 0.016 and 2.28 Pa.s*^n^*, and the *n* varied from 0.74 to 1.0, confirming the pseudoplastic behavior for solutions with Ch > 0.2% (*n* < 1). These values are in agreement with that determined by Wu et al. [18]: *K* = 0.17 Pa.s*^n^* and *n* = 0.75 for Ch solutions (2%) prepared with Ac (1%). The variation of these properties as a function of the Ch, for the three studied T, can be observed in Figure 5A,C. For all studied T, *K* increased following an exponential (Equation (5)) equation, while *n* decreased linearly (Equation (1)) as a function of Ch with R^2^ > 0.98 for both properties (Table 2). These same trends were observed when these properties were analyzed as a function of the pH of Ch solutions, certainly because of the linear relationship between pH and Ch (Table 2).

*K* = *A* e^(*B*[Ch])^ (5)

where *A* and *B* are empirical constants.

Hwang and Shin [27], working at 25 °C, increased Ch from 1 to 2.5% and calculated *K* values from 3.8 to 61.5 Pa.s*^n^*, which were higher than those in this work (Figure 5A). Nevertheless, fitting their data of *K* vs. Ch allowed us to observe that *K* increased exponentially with Ch, as in this study. Conversely, these authors calculated values of *n* from 0.52 to 0.48, which are quite inferior to those observed in this study, but also, *n* decreased linearly with Ch, similar to what was observed in this study (Figure 5C). These differences must be explained by the fact that a low viscosity Ch was used in this work. For Abu-Jdayil et al. [14], the relation between *K* and Ch followed a power law model, with *K* increasing as a function of Ch. In addition, Tovar et al. [22] observed that *K* and *n* values increased and decreased, respectively, with the increasing of Ch, at 20 °C, similar to that observed by Torres et al. [24]. Then, Ch solutions became more pseudoplastic with increasing Ch [27].

It can be observed in Table 2 that the T influenced the effect of Ch on both *K* and *n*: the parameters *B* and *A* of Equations (5) and (1), respectively, reduced with the increasing of T, although the effect of T on *K* can be better studied applying an Arrhenius equation type (Equation (6)).

*K* = *A* e^(Ea/RT)^ (6)

where R is the universal gas constant (8.314 kJ/(mol.K)), T is the absolute temperature (*K*), *A* is a pre-exponential constant, and Ea is the activation energy, defined as the energy required for the fluid molecule to move freely [15].

Thus, Ea was calculated after non-linear regression (R^2^ > 0.99) of *K* vs. 1/T for each study of Ch. The Ea stayed around 33 kJ/mol for Ch between 1.0 and 2.5%, dropping drastically to 5.5 kJ/mol when Ch decreased to 0.5%. The lowest Ea value for Ch = 0.5% can be due to the small intermolecular entanglement, which in turn corresponds to less restriction in the freedom of movement of the biopolymeric chains [21]. The values calculated in this work were similar to those calculated by El-Hefian et al. [21], who studied the effect of T on apparent viscosity of a solution with 10 g of Ch/L and calculated Ea = 20.9 kJ/mol, and similar to Bertolo et al. [15], who calculated the Ea from the variation of apparent viscosity of FFS based on blends of Ch/gelatin as a function of T, varying between 15.9 and 39.3 kJ/mol.

##### Effect of Acetic Acid Concentration on Rheological Properties

To the knowledge of the authors of this paper, so far there have not been sufficient studies of the effect of Ac on the rheological properties of Ch solutions. Benoso et al. [12] studied the effect of the pH (3.5–6.0) on Ch/gelatin blends solutions but not of a pure Ch solution. Thus, this can be considered as a first step in this field. All studied Ch solutions presented a shear-thinning (pseudoplastic) non-Newtonian behavior (Figure 4B,D,F), i.e., the Ac did not provoke any important change of fluid behavior. Nevertheless, the effect of Ac on the flow curves position in Figure 4B,D,F is still unclear, and a logical order was not observed. This can be better observed by analyzing the effect of Ac on *K* and *n*, calculated using Equation (4), as described in Section 3.2.1.

The first effect of Ac on rheological properties was that these values presented a narrower variation: *K* and *n* varied from 0.29 to 2.22 Pa.s*^n^*, and from 0.68 to 0.85, respectively (Figure 5B,D). Benoso et al. [12] analyzed solutions based on blends of Ch and two types of gelatin (GeA and GeB) as a function of pH (3.5–6) and observed that for Ch/GeA blend, *K* and *n* varied between 8 and 9.9 mPa.s*^n^* and between 0.94 and 0.97, respectively, which can be also considered a very narrow variation. The second effect was that *K* increased as a function of Ac for lower Ac concentrations and decreased for higher Ac, showing a parabolic positive behavior (Figure 5B,D). This same behavior, but in the inverse sense, was observed with *n*. The parameters of parabolic equations used to calculate the data corresponding to dotted lines plotted in Figure 5B,D were presented in Table S2.

Rinaudo et al. [36] studied the effect of Ac on the relative viscosity (µ_rel_ = µ/µ_0_ with µ_0_ being the viscosity of the solvent used to prepare the solution) and observed that µ_rel_ increased in the presence of acid, reaching a maximum at different acid concentrations depending on the biopolymer concentration, similar to what was observed in Figure 5B,D. These authors saw that the maximum in µ_rel_ was very broad and remained nearly constant in a large range of initial acid concentration. Moreover, the low *K* value for Ac = 0.2% can also be associated with the low solubilization of Ch, as demonstrated by SEM (Section 3.1.1) and XRD (Section 3.1.3), whereas the low value for Ac = 1% should be associated with the high ionic strength occurring with a high acid concentration [34], since salts were also detected in freeze-dried Ch solutions prepared with high Ac (Figure 3).

Benoso et al. [12] observed that *K* and *n* increased linearly as a function of pH for solutions based in blends Ch/GeA, while for Ch/GeB, these parameters increased exponentially. This demonstrates the complexity of the effect of the acid concentration, and consequently, the pH, on rheological properties of colloidal solutions. Probably due to this complexity, Ea, calculated as explained in 3.2.1.1, varied without a logical behavior with Ac. In fact, Ea values were higher for 0.6% (49 kJ/mol) followed by Ch solutions with 0.2 and 1%, with the same value (42 kJ/mol), and for 0.4 and 0.8%, also with the same value (30 kJ/mol). Further studies are necessary to confirm and explain this behavior.

#### 3.2.2. Dynamic Tests: Frequency Sweep

Besides it being interesting to study macromolecular relaxation phenomena, viscoelastic properties are also useful in examining gravity driven phenomena such as coating and sagging, being a valuable tool in product development [33]. In dynamic tests, the main viscoelastic properties are the storage (G’) and loss (G”) moduli which allow the calculation of tanδ = G”/G’ [30]. In this work, these properties were mainly determined by frequency sweep tests. Temperature scanning tests were not performed because this system does not show thermal transitions [38].

Classically, tests for strain sweep were previously performed to define the LVR [33]. Thus, 5% of strain was taken as sufficient good value to produce reproducible data staying inside of the LVR. This strain value was the same used by Bertolo et al. [15] and Benoso et al. [12], but it was higher than that selected by Tovar et al. [20] (0.4%) and by Wu et al. [18] (0.2%) and lower than that chosen by Horn et al. [16] (10%), both working on viscoelastic properties of Ch-based solutions.

##### Effect of Chitosan Concentration on Viscoelastic Properties

**Figure 6:**
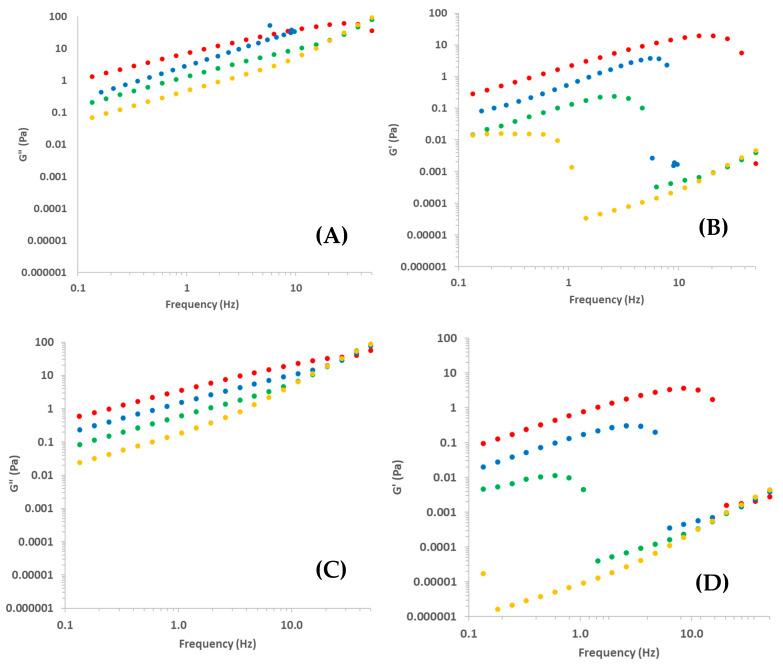
Loss (G”, **A**,**C**) and storage (G’, **B**,**D**) moduli determined by frequency sweep tests of chitosan solutions prepared with several chitosan concentrations: 2.5 (red), 2.0 (blue), 1.5 (green), 1.0 (yellow); at 25 (**A**,**B**) and 45 °C (**C**,**D**).

The variation of G’ and G” as a function of the frequency at 25 and 45 °C is shown in Figure 6.

No data on the Ch solution with Ch = 0.5% were plotted because both properties were very low and out of the rheometer sensibility. Overall, G” > G’ for all studied frequencies, Ch and T, always with Tanδ >> 1, agreeing with observations by Martínez-Ruvalcaba et al. [26], Horn et al. [14], and Tovar et al. [22]. This means that Ch solutions behaved like a sol, as could be expected from steady state results. According to Steffe [33], very high values of Tanδ are expected for dilute solutions. In physical chemistry of macromolecules, dilute chitosan solutions with Ch ranging from 0.01 to 0.1% are usually studied for determinations of intrinsic viscosity [39]. Certainly because of this, Martínez-Ruvalcaba et al. [26] analyzed Ch solutions with Ch = 0.5–2.0%, considering them as concentrated solutions. Nevertheless, according to the linear viscoelastic theory [30], these biopolymers-based solutions must be considered as diluted ones (tanδ >> 1).

According to Steffe [33], materials usually exhibit more solid-like characteristics at higher frequencies. This means that a G” and G’ crossover would be expected around high frequencies, as observed by Torres et al. [24] studying gels based in Ch crosslinked with glutaraldehyde. Nevertheless, this phenomenon was not observed in this study.

The effect of Ch and T on these curves were evident (Figure 6). For a constant frequency, the increasing of Ch increased both G’ and G”, due to the entanglements of Ch chains, and the increasing of T decreased both moduli, due to the positive effect of T on macromolecular motion, as explained previously. These behaviors agree with those observed by Martínez-Ruvalcaba et al. [26].

Regarding the solid-viscoelastic character of Ch solutions, it is interesting to observe that a sudden drop occurred in G’ curves with the increasing of the frequency, and the position of these drops depended on Ch. This drop may have occurred because, at high frequencies, these solutions lost the continuity in the biopolymeric chain, moving out of the LVR. The critical frequency (*f*_cr_) where these drops occurred was calculated by fitting linear equations to data in domains just before and after the drop and calculating the interception between these equations, for each curve. All linear regressions presented coefficient correlations higher than 0.94, calculated using the software Excel 2020^®^.

**Figure 7:**
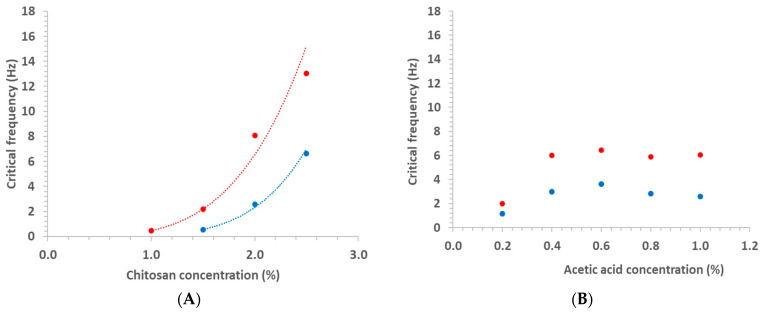
Variation of the critical frequency for G’ drop as a function of chitosan concentration (**A**) and acetic acid concentration (**B**), at 25 (red) and 45 °C (blue).

Then, it was possible to observe (Figure 7A) that this critical frequency increased with Ch according to a power law equation (Equation (7)). It was not possible to calculate *f*_cr_ for Ch solution with Ch = 1% at 45 °C because the curve began in the drop domain.

*f*_cr_ = A[Ch]^B^ (7)

where *f*_cr_ is the critical frequency, and A and B are empirical parameters calculated by non-linear regression using the software Excel 2020^®^ (Table 2). The higher values of A at 25 °C confirm the higher values of *f*_cr_ at this temperature, and the higher value of B at 45 °C inform that the effect of Ch was more important at this temperature.

Because of this behavior of G’, Ch solutions were characterized by their G” curves. To analyze these curves, another power law model (Equation (8)) was considered [25].

G″ = α ω^n″^ (8)

where α is a pre-potential constant, ω is the angular frequency (rads/sec), and n” is the so-called relaxation exponent, which is a characteristic property of macromolecular chains arrangement: n” = 0.5 means that the system is in the sol–gel transition, n” < 0.5 means that it behaves as a viscoelastic solid, and n” > 0.5 means that it behaves as viscoelastic liquid [25].

**Figure 8:**
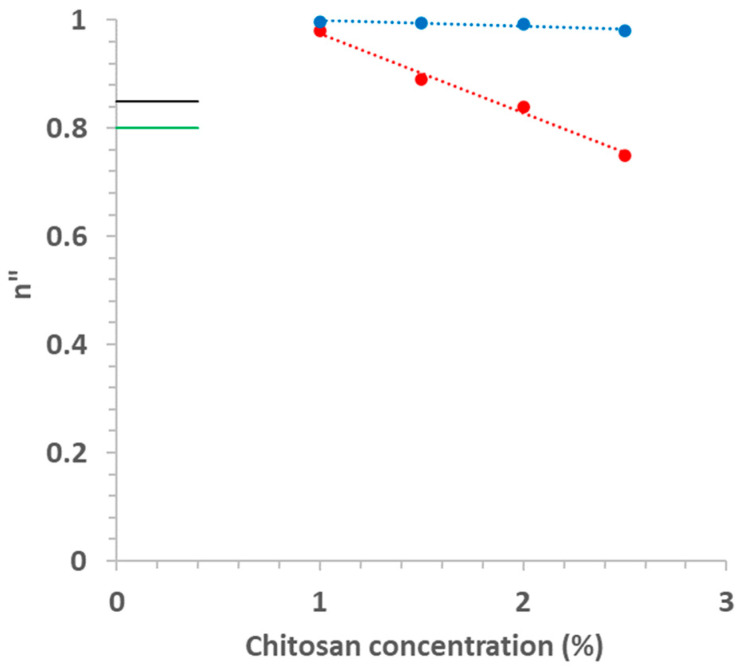
Relaxation exponent (n”) calculated with Equation (8) as a function of chitosan concentration at 25 (red) and 45 °C (blue). Lines green and black close to the Y-axis indicate the average values of n” for chitosan solutions prepared with different acetic acid concentrations at 25 (green) and 45 °C (black).

Equation (8) was fitted by non-linear regression (R^2^ > 0.97) to G” vs. angular frequency (in rad/s) data allowing to confirm that all Ch solutions behaved as viscoelastic liquid, with n” > 0.7, and that n” increased linearly (Table 2) as a consequence of the reduction of Ch in a more important manner for data calculated at 25 °C (Figure 8). Similar behavior was observed by Rwei et al. [25]. Besides depending on the concentration, n” also depends on the biopolymer molecular weight [25] and on T, as observed in Figure 8: increasing T from 25 to 45 °C reduced drastically the effect of Ch on n” due to the increase in Ch chain mobility [21], reducing the effect of biopolymer–biopolymer interactions [28].

##### Effect of Acetic Acid Concentration on Viscoelastic Properties

**Figure 9:**
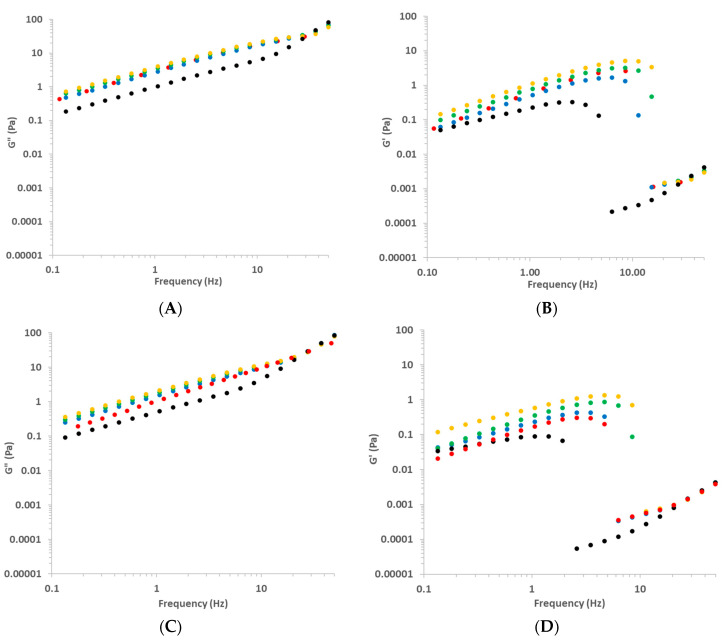
Loss (G”, **A**,**C**) and storage (G’, **B**,**D**) moduli determined by frequency sweep tests of chitosan solutions prepared with several acetic acid concentrations: 2.5 (red), 2.0 (blue), 1.5 (green), 1.0 (yellow); at 25 (**A**,**B**) and 45 °C (**C**,**D**).

The variation of G’ and G” for Ch solution as a function of frequency for all studied Ac can be observed in Figure 9. In addition, in this case, it was observed that G” was always higher than G’ (tanδ >> 1), and that G’ also dropped with the increasing of the frequency (Figure 9), as observed in Section 3.2.2. Nevertheless, a logical effect of Ac was not observed in these results even if the effect of T was as expected. Martínez-Ruvalcaba et al. [24] prepared Ch solutions with 1% of acetic and lactic acids and HCl and observed lower values of both moduli for solutions with HCl, which must have lower pH.

The dependence of the *f*_cr_ on Ac was less evident (Figure 7B). At 25 °C, *f*_cr_ stayed around 6.1 Hz for Ac between 1 and 0.4%, dropping to 2 Hz when Ac was reduced to 0.2%, while for 45 °C, *f*_cr_ dropped from 3 to 1.2 Hz when Ac decreased from 1–0.4 to 0.2%. The values at 45 °C were lower than that at 25 °C, similar to that observed previously (3.2.2.1). Again, Equation (8) was fitted to data [G” = f(ω)], allowing us to observe an important effect of Ac on n” (Figure 8): n” stayed around 0.80 and 0.85 at 25 and 45 °C, respectively.

## 4. Conclusions

The results obtained in this study evidenced the importance of the definition of the acetic acid concentration when preparing chitosan solutions. Very low or very high acid concentration can provoke unexpected changes in the solution′s rheological behavior properties that is different from the effect of the chitosan concentration. In this last case, rheological properties can increase or decrease as a function of Ch but always linearly. This same behavior was not observed regarding the effect of Ac.

The consistency index (*K*) increased as a function of chitosan concentration following an exponential behavior, while the index flow behavior (*n*) decreased linearly. The activation energy, calculated from the variation of the consistency index as a function of the temperature, was high for solutions with a concentration of 1% or more and low for solutions with 0.5% concentration.

The effect of acetic acid concentration was less evident when compared with that of the Ch effect and not necessarily observed by other authors. No logical effect was observed on flow curves position as a consequence of acetic acid concentration. The consistency index (*K*) and flow behavior (*n*) varied parabolically, in a positive and negative manner, respectively, as a function of chitosan concentration, where both maximum and minimum values persisted in a large range of concentration. Moreover, the effect of temperature, calculated as the activation energy from the consistency index, showed a behavior impossible to explain with current data. The activation energy was higher for solutions with 0.6% of chitosan and lower for solutions with 0.4 and 0.8%, thus more research is needed to explain this effect.

Regarding the viscoelastic properties, it can be concluded that all studied solutions, irrespective to chitosan and acetic acid concentrations, behaved as diluted solutions, with G” > G’ (tanδ >> 1), without showing any tendencies of crossover at high frequency. Contrarily, a sudden drop in G’ was observed when the frequency increased for all samples. These drops occurred at low frequency values when chitosan concentration decreased but were insensitive at acetic acid concentrations higher than 0.2%. As these solutions were diluted, in linear viscoelastic theory sense, the increasing of frequency provoked discontinuity of macromolecular structure, falling out of the linear viscoelasticity region.

A power model was fitted to G” curves allowing the calculation of the relaxation exponent, which was always higher than 0.5, confirming that these systems behave as a viscoelastic liquid. This relaxation exponent increased linearly when chitosan concentration decreased, and it did so in a more important manner as 25 °C. On the other hand, this property was insensitive to acetic acid concentration, being slightly higher at 45 °C.

## Figures and Tables

**Table 1:** Examples of different preparation conditions for chitosan and film-forming solutions.

Material as Described by Authors	Chitosan Concentration	Acid Solutions	References
Commercial chitosan, medium Mw, deacetylation: 75–85%, viscosity: 200–800 cP	1%	1% of acetic acid	[1]
Commercial chitosan, deacetylation: 75%, viscosity: 150–500 mPa.s	---	6% of acetic acid	[2]
Commercial chitosan, higher molecular weight (Mw)	2.5%	1% of acetic acid	[3]
Commercial chitosan, Mw = 180 kDa, deacetylation: 85%	4%	4% of acetic acid	[4]
Commercial chitosan, medium Mw, deacetylation: 75–85%	3%	1% of acetic acid	[5]
Commercial chitosan, viscosity: 800–2000 cP	1%	1% of acetic acid	[6]
Commercial chitosan, Mw = 110 kDa, deacetylation: ≥75%	1.25%	1% of acetic acid	[7]
Commercial chitosan, Mw = 100–3000 kDa	0.01%	0.1% of acetic acid	[8]
Commercial chitosan, medium Mw	1%	1% of acetic acid	[9]
Commercial chitosan, Mw = 100–3000 kDa, deacetylation: 82%	6.7%	10 M of acetic acid	[10]
Commercial chitosan, Mw = 50–190 kDa, deacetylation: 75–85%	1.5%	2% of acetic acid	[12]
Commercial chitosan, Mw = 190–310 kDa, deacetylation: 75–85%, viscosity: 200–800 Cp	---	pH = 4.5 with HCl	[13]
Commercial chitosan, Mw = 8–12 kDa	0.1–3%	1% of acetic acid	[14]
Chitosan from *Doryteuthis* spp. produced in laboratory scale, Mw = 327 kDa, deacetylation: 6.7%	1%	1% of acetic acid	[15]
Chitosan from *Loligo* sp. produced in laboratory scale, Mw = 428 kDa, deacetylation: 9.05%	1%	1% of acetic acid	[16]
Chitosan from blue crab (*Callinectes sapidus*) shells and tongs, produced in laboratory scale, Mw = 57.4 kDa, deacetylation: 80.8%	2%	2% of lactic acid	[17]
Commercial chitosan, Mw = 98.72 kDa, deacetylation: 92%	2%	1% of acetic acid	[18]
Commercial chitosan, Mw = 190–310 kDa, deacetylation: 75%	1.5 and 2%	1% of acetic acid	[19]
Commercial chitosan, Mw = 71 (low), 220 (medium), and 583 kDa (high); deacetylation: 85–90%	2%	1% of acetic acid	[20]
Commercial chitosan, deacetylation: 88.1%	2–10 g/L	0.1 M acetic acid	[21]
Commercial chitosan, Mw= 140 kDa, deacetylation: 90.7%	1–3%	acetic or lactic acids	[22]
Chitosan from shrimp shells produced in laboratory scale, Mw = 213 kDa, deacetylation: 99.7%	0.25–0.75%	3% of acetic or 1% of lactic acids	[23]
Commercial chitosan, deacetylation ≥ 85%	2.5%	3% of acetic acid	[24]
Commercial chitosan, Mw = 200 kDa, deacetylation: 88%	5–12%	2.5–7.5% of acetic acid	[25]
Commercial chitosan, Mw = 108 kDa, deacetylation: 25%	1–5	1% of acetic, lactic acids or HCl	[26]
Chitosan from *Chitinonecetes opilio* produced in laboratory scale, Mw = 171 kDa, deacetylation: 91%	0.25–2.5%	0.1 M NaCl/0.1 M acetic acid	[27]

**Table 2:** Parameters calculated by fitting Equations (1), (5), (7) and (8) to data: Ch and Ac are chitosan and acetic acid concentrations, respectively; *K* is the consistency index; *n* is the flow behavior; *f*_cr_ is the critical frequency; and n” is the relaxation exponent.

Temperature (°C)	A	B	R^2^
*Y* = pH and *X* = [Ch] Equation (1)			
25	3.55	0.44	0.974
*Y* = pH and *X* = [Ac] Equation (1)			
25	6.19	−1.82	0.978
*Y* = *K* and *X* = [Ch] Equation (5)			
25	7.4 × 10^−3^	2.35	0.998
35	6.4 × 10^−3^	2.21	0.997
45	5.7 × 10^−3^	2.05	0.999
*Y* = *n* and *X* = [Ch] Equation (1)			
25	1.06	−12.2 × 10^−2^	0.977
35	1.04	−10.6 × 10^−2^	0.994
45	1.04	−9.4 × 10^−2^	0.984
*Y* = *f*_cr_ and *X* = [Ch] Equation (7)			
25	0.47	3.80	0.958
45	0.08	4.94	0.996
*Y* = n” and *X* = [Ch] Equation (8)			
	α	n”	
25	1.00	−0.01	0.795
45	1.12	−0.15	0.988

## Data Availability

Data is available under request to the correspondent author.

## Supplementary Materials

The following supporting information can be downloaded at https://www.mdpi.com/article/10.3390/foods11172692/s1, Figure S1: Chitosan solutions prepared with 2.5, 2.0, 1.5, 1.0, and 0.5% of chitosan (from left to right) and 1% of acetic acid; Figure S2: Chitosan solutions prepared with 2% of chitosan and 1, 0.8, 0.6, 0.4, 0.2, and 0% (water) of acetic acid (from left to right); Table S1: Values of the consistency index (*K*), the index flow behavior (*n*), and correlation coefficient (R2) calculated by fitting Equation (4) to flow curves data by non-linear regression; Table S2: Constants of polynomial equation (*K* or *n* = A[Ac]^2^ + B[Ac] + C) calculated by non-linear regression.
