# Supplementary material for: Rheological and Viscoelastic Properties of Chitosan Solutions Prepared with Different Chitosan or Acetic Acid Concentrations
Source: Foods. 2022 Sep 3;11(17):2692. doi: 10.3390/foods11172692 (PMC9455163; doi:10.3390/foods11172692)
Supplement: Supplementary file 1 [file foods-11-02692-s001.zip › foods-1792494-supplementary.pdf]

foods

MDPI

Supplementary Materials

Rheological and Viscoelastic Properties of Chitosan Solutions Prepared with Different Chitosan or Acetic Acid Concentrations

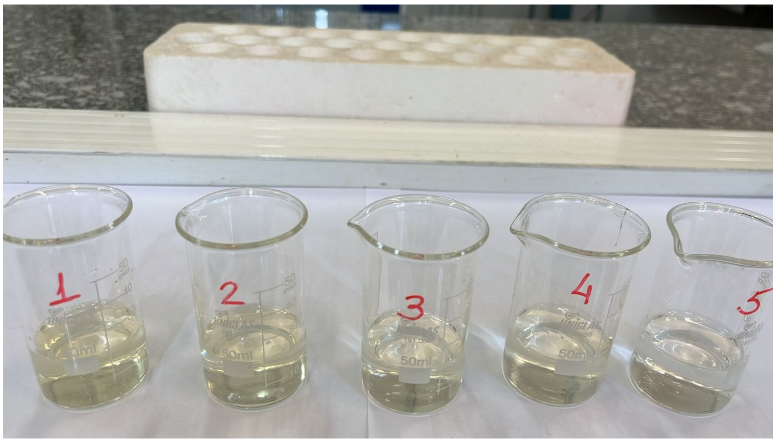

Figure S1. Chitosan solutions prepared with 2.5, 2.0, 1.5, 1.0 and 0.5% of chitosan (from left to right), and 1% of acetic acid.

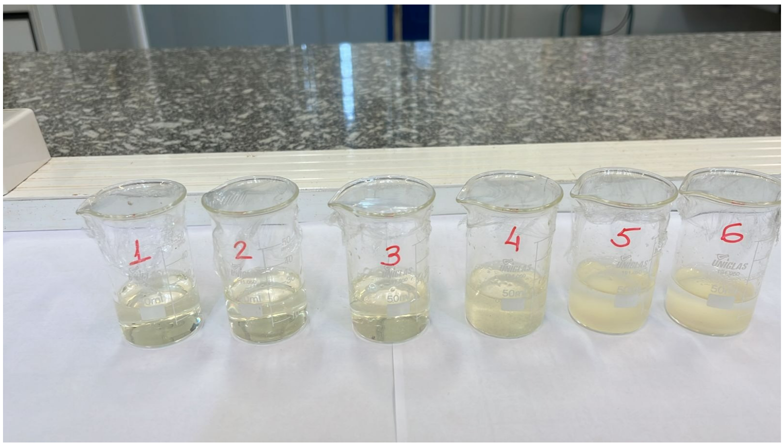

Figure S2. Chitosan solutions prepared with 2% of chitosan and 1, 0.8, 0.6, 0.4, 0.2 and 0% (water) of acetic acid (from left to right).

Table S1. Values of the consistency index (*K*), the index flow behavior (*n*), and correlation coefficient (R^2) calculated by fitting Equation 4 to flow curves data by non-linear regression.

Temperature (°C)	Chitosan Concentration (%)	*K* ($Pa.s^n$)	*n* (-)	R^2
25	0.5	0.0181	1.025	0.999
	1.0	0.0997	0.918	0.999
	1.5	0.3066	0.880	0.998
	2.0	0.7762	0.832	0.994
	2.5	2.2804	0.738	0.997
35	0.5	0.0166	0.988	0.999
	1.0	0.0653	0.930	0.999
	1.5	0.2047	0.888	0.999

	2.0	0.5491	0.837	0.996
	2.5	1.4540	0.769	0.997
45	0.5	0.0157	0.904	0.987
	1.0	0.0449	0.929	0.999
	1.5	0.1280	0.903	0.997
	2.0	0.3553	0.854	0.995
	2.5	0.9460	0.802	0.997
	Acetic acid concentration of (%)			
25	0.2	0.8372	0.744	0.995
	0.4	1.5257	0.722	0.990
	0.6	2.2185	0.682	0.987
	0.8	1.0341	0.767	0.986
	1.0	0.9371	0.790	0.984
35	0.2	0.4915	0.767	0.988
	0.4	0.1.173	0.735	0.989
	0.6	1.1743	0.735	0.987
	0.8	0.8029	0.769	0.989
	1.0	0.7000	0.799	0.995
45	0.2	0.2911	0.795	0.995
	0.4	0.7078	0.763	0.989
	0.6	0.6363	0.779	0.985
	0.8	0.4792	0.806	0.992
	1.0	0.3202	0.848	0.985

Table S2. Constants of polynomial equation (K or n = A[Ac]^2 + B[Ac] + C) calculated by non-linear regression.

Temperature (ºC)	A	B	C	R^2
K				
25	-6.16	7.24	-0.33	0.655
35	-3.47	4.18	-0.12	0.746
45	-2.21	2.57	-0.08	0.817
n				
25	0.39	-0.39	0.81	0.750
35	0.32	-0.32	0.81	0.855
45	0.29	-0.27	0.83	0.969
